# A comparative analysis of diffusion-weighted imaging and ultrasound in thyroid nodules

**DOI:** 10.1186/s12880-019-0381-x

**Published:** 2019-11-21

**Authors:** Weidan Kong, Xiuhui Yue, Jiliang Ren, Xiaofeng Tao

**Affiliations:** Department of Radiology, Shanghai Ninth People’s Hospital, Shanghai Jiao Tong University School of Medicine, Shanghai, China

**Keywords:** Thyroid nodules, Ultrasound, Diffusion-weighted imaging

## Abstract

**Background:**

Diffusion-weighted imaging (DWI) and ultrasound are commonly used methods to examine thyroid nodules, but their comparative value is rarely studied. We evaluated the utility of DWI and ultrasound in differentiating benign and malignant thyroid nodules.

**Methods:**

A total of 100 patients with 137 nodules who underwent both DWI and ultrasound before operation were enrolled. The T1 and T2 signal intensity ratio (SIR) of each thyroid nodule was calculated by measuring the mean signal intensity divided by that of paraspinal muscle. The apparent diffusion coefficient (ADC) value and the SIR of benign and malignant thyroid nodules were analyzed by two-sample independent *t* tests. The sensitivity, specificity, and accuracy of DWI and ultrasound were compared with chi-square tests.

**Results:**

There was no significant difference in the SIR between benign and malignant thyroid nodules. The ADC value was significantly different. At the threshold value was 1.12 × 10^− 3^ mm^2^/s, the maximum area under the curve was 0.944. The sensitivity, specificity, and accuracy were 84.9, 92.2, and 87.6% respectively. The corresponding values of ultrasound diagnosis were 90.1, 80.4, and 86.9%.

**Conclusions:**

Ultrasound has high sensitivity in differentiating benign and malignant thyroid nodules, and the ADC value has high specificity, but there is no statistical difference in sensitivity or specificity between the two modalities. DWI and ultrasound each have their own advantages in differentiating benign and malignant thyroid nodules.

## Background

The incidence of thyroid malignant tumors has increased significantly in the past decades. The detection rate in healthy people ranges from 16 to 67% [1]. Most of the nodules tend to have a benign nature and differentiated thyroid cancer (mainly papillary and follicular carcinomas) accounts for the majority of cases, however,the malignancy has been reported in less than 5% [2] of the nodules. If early detection and follow-up active treatment are carried out, the 10-year survival rate can be as high as 90% [3]. Ultrasound is the preferred method for thyroid tumor diagnosis. It has good diagnostic ability to detect morphological manifestations, calcification, and cystic necrosis of thyroid lesions. However, previous studies have shown that diagnostic results are susceptible to the operator’s influence [4]. Diffusion-weighted imaging (DWI) has become a popular modality for identifying thyroid nodules in recent years. Some researchers have suggested that the T2 signal intensity ratio (SIR) of thyroid nodules can be used as a quantitative parameter to differentiate benign from malignant nodules, which is of great significance for preoperative diagnosis [5]. The purpose of this study was to explore the best diagnostic threshold of DWI in differentiating benign and malignant thyroid nodules, and to compare the diagnostic value of DWI with ultrasound.

## Methods

### Patients

The Institutional Review Board of Shanghai Ninth People’s Hospital approved this retrospective study, and the requirement for informed consent was waived. The following criteria were adopted for patient selection: 1) primary thyroid tumor; 2) patients underwent both conventional DWI and ultrasound scan before treatment; 3) masses with short axis ≥ 10 mm; 4) magnetic resonance (MR) images could be acquired and interpreted. Through a comprehensive search of our institutional medical report database from July 2017 to January 2019, we identified 100 patients (mean age, 49 years; range, 23–79 years) with 137 nodules (mean short axis, 18 mm; range, 5–76 mm). The final diagnoses based on histopathological results in 137 nodules, and the diagnostic results of DWI and ultrasound were compared.

### Image acquisition

#### DWI examination

All MR imaging (MRI) examinations were performed on a 3.0 T scanner (Philips Ingenia 3.0 T; Amsterdam, the Netherlands). The head and neck coils were placed over the thyroid surface. Patients were placed in a supine position with their neck, back, shoulders relaxed and instructed to breathe smoothly and avoid swallowing.

The MRI acquisition parameters were: T1WI turbo spin echo (TSE) (repetition time [TR] = 450 ms, echo time [TE] = 20 ms, thickness = 3 mm, gap = 1 mm, field of view [FOV] = 240 mm × 240 mm, matrix = 300 × 240; T2WI DIXON-TSE (TR = 2500 ms, TE = 100 ms, thickness = 3 mm, gap = 1 mm, FOV = 240 mm × 240 mm, matrix = 300 × 240); DWI (TR = 400 ms, TE = 70 ms, thickness = 3 mm, gap = 1 mm, FOV = 240 mm × 240 mm, matrix = 220 × 180). The b values were 0 and 1000 s/mm^2^.

#### Ultrasound examination

All ultrasound examinations were performed on a ultrasound scanner (Toshiba-Aplio 400; Tokyo, Japan). The probe frequency was 5–12 MHz. During examination, the patient was in a supine position, with the head slightly backward to fully expose the neck. The probe was placed over the thyroid region. The nodule boundary, echo, blood flow, calcification, and peripheral lymph node enlargement were observed.

### Image analysis

Apparent diffusion coefficient (ADC) measurements were made by two radiologists with 3 and 7 years of experience in head and neck imaging, who were blinded to the clinical information and diagnosis. DWI image analysis was performed on a Philips post-processing workstation to measure the average ADC value of each nodule (b value 0, 1000 s/mm^2^) and the SIRs on T1WI and T2WI. For the ADC value, a region of interest (ROI) of the same size on different sections was selected for multi-point measurement, and the average value was obtained. The ROIs were selected while avoiding cystic degeneration, hemorrhage, and necrosis as much as possible. The SIR was calculated for each sequence as a ratio of signal intensity of the thyroid nodule to that of the paraspinal muscle.

Ultrasound examination was performed by a radiologist with 7 years of experience in head and neck imaging, who was blinded to clinical information and diagnosis. The Thyroid Imaging Reporting AND Date System (TI-RADS) classification method was used in the ultrasound diagnosis report, which stipulated that grade 1–3a was benign and grade 3b–5 was malignant. Finally, each nodule was given a grade and qualitative diagnosis.

### Statistical analysis

SPSS 20.0 software (IBM Corp., Armonk, NY) was used for statistical analysis. Differences in ADC, T1SIR, and T2SIR in patients with benign and malignant thyroid nodules were evaluated by independent sample t tests. Difference were considered statistically significant when *P* < 0.05. The ADC thresholds for differentiating benign and malignant thyroid nodules were obtained from receiver operating characteristic (ROC) curves. Finally, the diagnostic values of ultrasound and DWI were compared by paired chi-square tests.

## Results

### Pathology

A total of 137 thyroid nodules were found in 100 patients (21 male and 79 female), including 79 in the left lobe, 51 in the right lobe, and 7 in the isthmus. There were 86 malignant nodules (82 papillary, 2 follicular, and 2 lymphoma) and 51 benign nodules (31 nodular goiters, 11 thyroid adenomas, and 9 Hashimoto’s thyroiditis).

### ADC and SIR

**Fig. 1:**
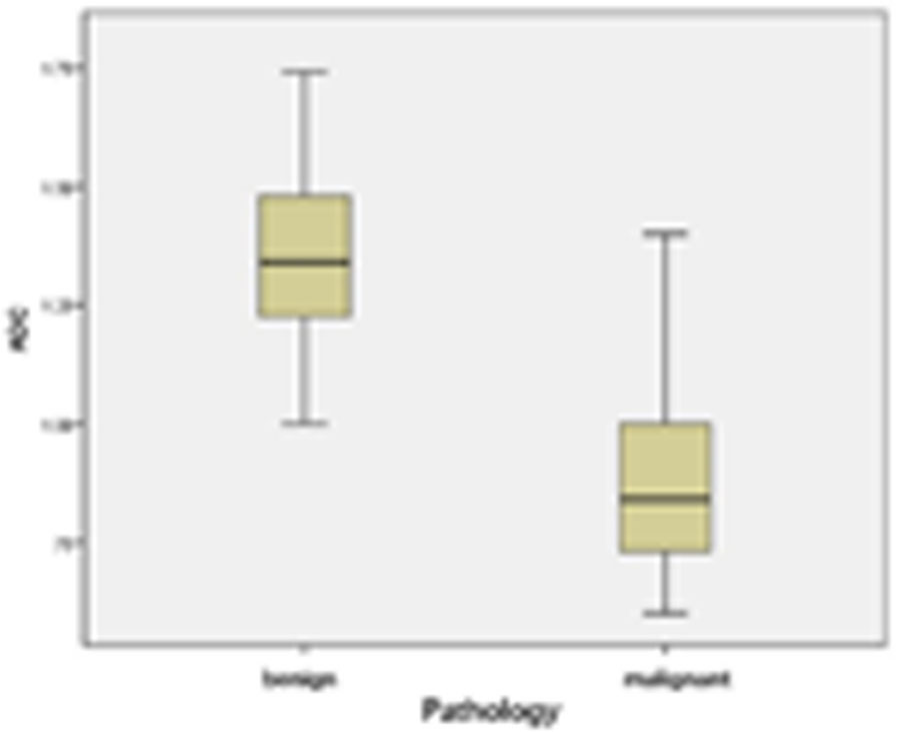
Box plot shows apparent diffusion coefficient (ADC) values for malignant and benign thyroid nodules. The bottom, middle, and top box lines indicate the 25th percentile, median, and 75th percentile, respectively. The error bars show the smallest and largest values in 1.5-box lengths of the 25th and 75th percentiles

**Fig. 2:**
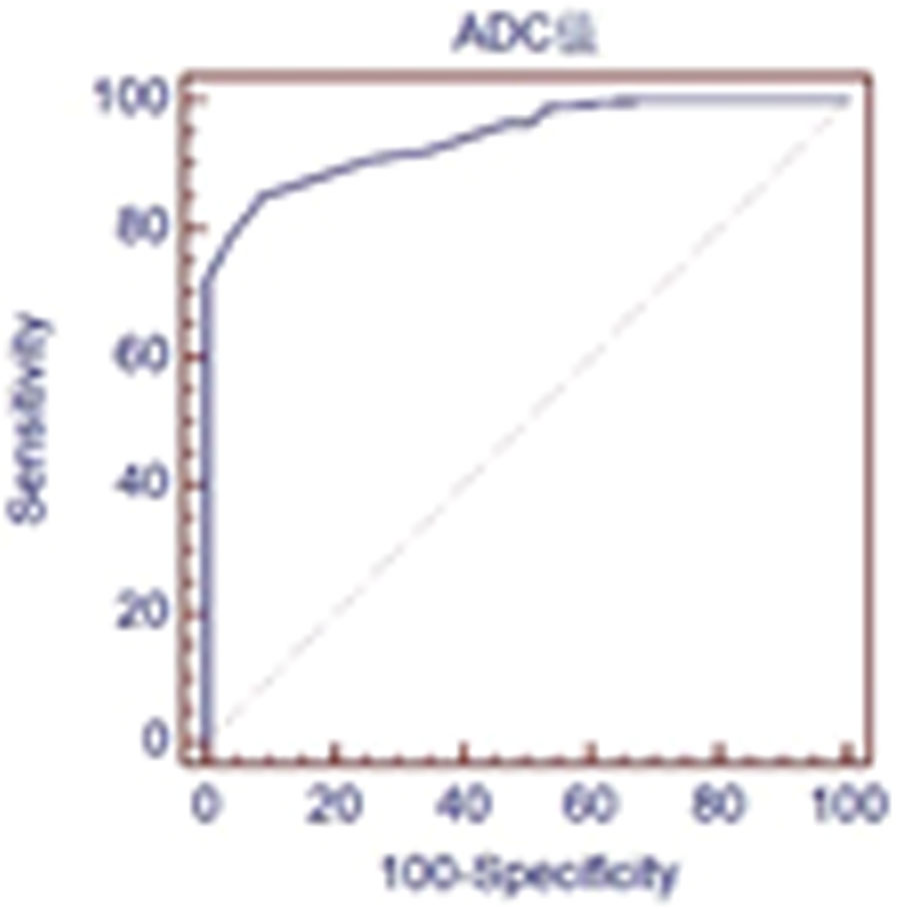
ROC curve of the ADC value used to differentiate between malignant and benign thyroid nodules (b = 1000 s/mm^2^). The maximum AUC is 0.994

The average ADC value of benign thyroid nodules (1.36 ± 0.17 × 10^− 3^ mm^2^/s) was higher than that of malignant thyroid nodules (0.89 ± 0.21 × 10^− 3^ mm^2^/s), and the difference was statistically significant (*P* < 0.05, Fig. 1). There were no significant differences in T1SIR or T2SIR between benign and malignant thyroid nodules (*P* > 0.05). According to ROC curve analysis, the best ADC threshold for differentiating benign and malignant thyroid nodules was 1.12 × 10^− 3^ mm^2^/s. The maximum area under the corresponding curve was 0.944, and the sensitivity and specificity were 84.9 and 92.2%, respectively (Fig. 2). The ADC values and T1 and T2 SIRs of benign and malignant thyroid nodules are detailed in Table 1.

**Table 1:** Comparison of ADC values and signal intensity ratios of T1 and T2 (SIR) in benign and malignant thyroid nodules

MR sequence	Benign nodule(*n* = 51)	Malignant nodule(*n* = 86)	*P* value
ADC	1.36 ± 0.17 (1.31~1.40)	0.89 ± 0.21 (0.84~0.93)	<0.05
T1SIR	1.77 ± 0.72 (1.57~1.97)	1.81 ± 0.57 (1.69~1.94)	0.42
T2SIR	1.80 ± 0.49 (1.66~1.94)	1.64 ± 0.41 (0.84~0.93)	0.07

Note: The data in the table are expressed by mean (+standard deviation) and the confidence interval range in brackets is 95%. *P* < 0.05 indicates that there is statistical difference

### Diagnostic results of DWI and ultrasonography

**Fig. 3:**
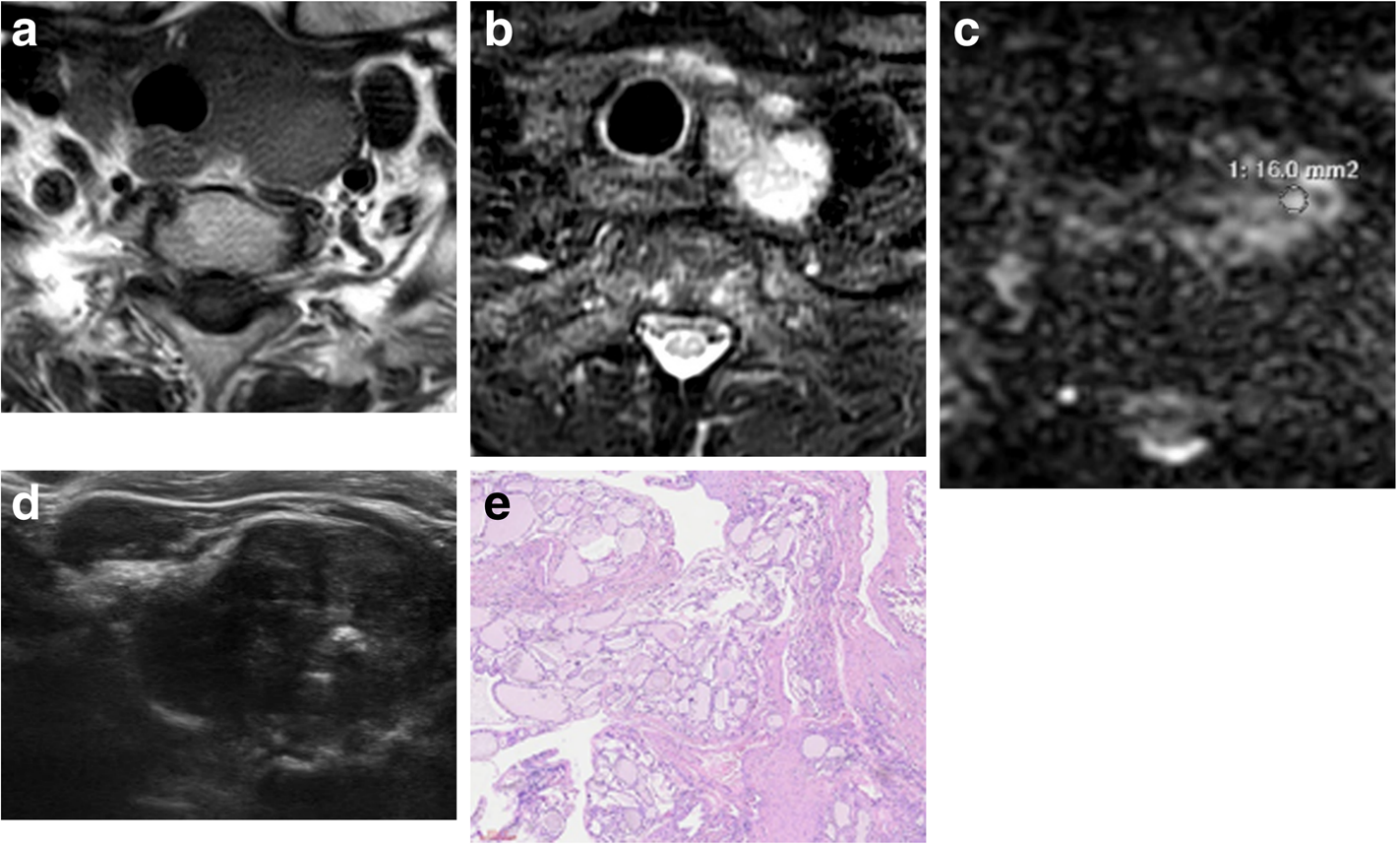
A 45-year-old man with papillary carcinoma of the left lobe of the thyroid (white arrow). **a**: axial T1WI showed low signal with multiple nodules in the left lobe, **b**: axial T2WI fat suppression on the same level showed high signal with unclear boundary, **c**: ADC map showed obvious high signal on the same level, and the ADC value is 1.0 × 10^− 3^ mm^2^/s, **d**: Ultrasound transverse section showed hypoechoic nodule in left thyroid lobe, irregular shape, unclear boundary, uneven echo, and multiple hyperechoic and hypoechoic points. TI-RADS:4c. **e**: HE staining (10*40) showed irregular and branching papillary in nodules, with packed arranged glassy cells with atypical nuclear, intranuclear inclusions and nuclear grooves were also observed, fibrous strips (pseudocapsule) were seen at the edge, and neoplastic cell nests infiltrated into the capsule

**Fig. 4:**
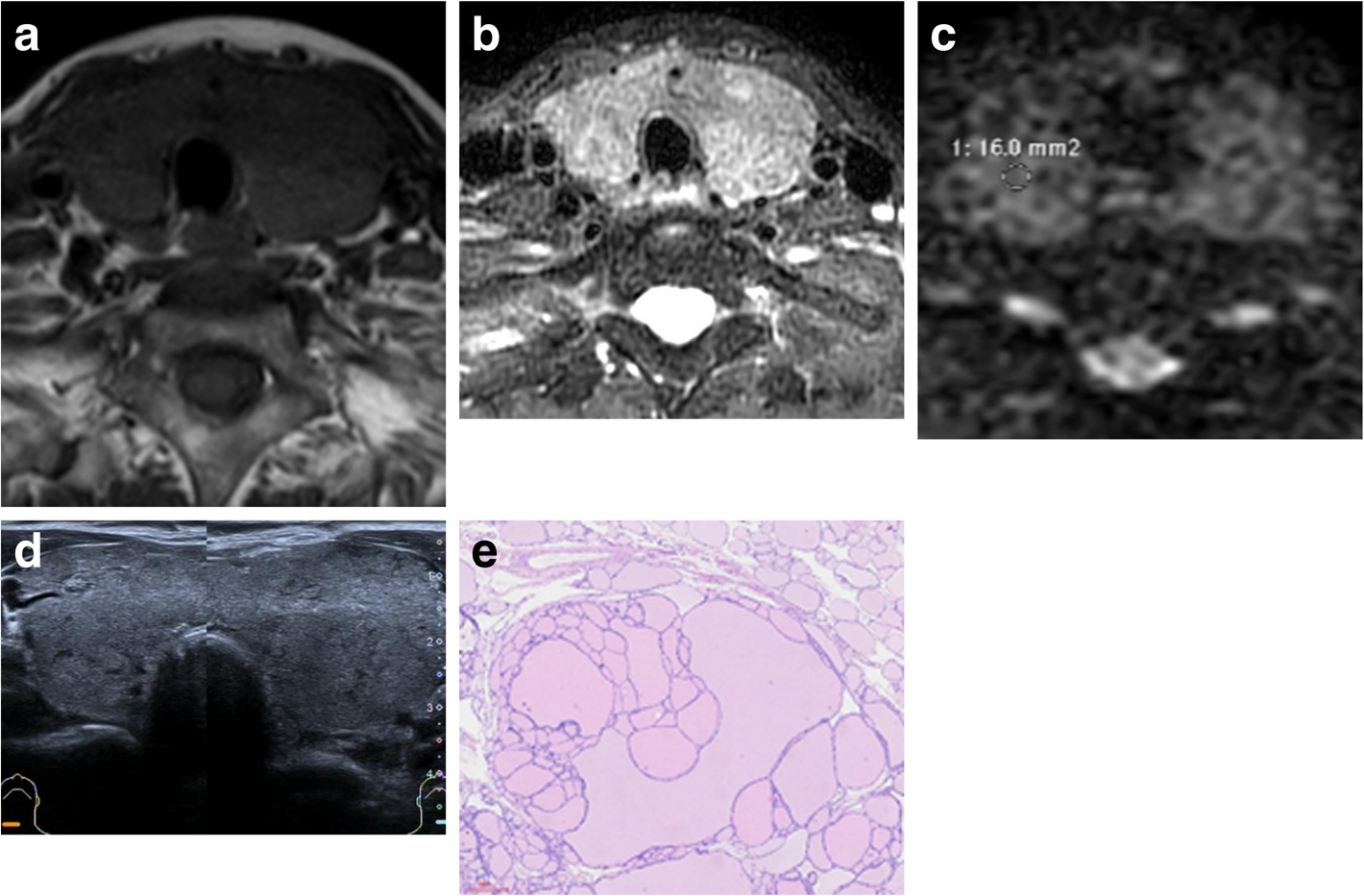
A 63-year-old woman with a bilateral nodular goiter of the thyroid (white arrow). **a**: axial T1WI showed bilateral lobe enlargement with low-signal shadows, **b**: axial T2WI fat suppression on the same level showed high signal with unclear boundary, **c**: ADC map showed obvious high signal on the same level, and the ADC value is 1.8 × 10^− 3^ mm^2^/s, **d**: Ultrasound transverse section showed that bilateral thyroid lobes were plump, isthmus was thicker, surface was smooth, echo was medium, thickening and uneven distribution, TI-RADS:3a. **e**:HE staining (10*40) showed that the tightly packed follicles were filled with colloid, some endocytosins of different sizes absorbed vacuolesv vacuoliation, and microvessels decreased between follicles

There were 82 papillary thyroid carcinomas (Fig. 3) and 3 follicular thyroid carcinomas. Most showed high or slightly high signals on DWI, and the signals were relatively uniform. Two lymphomas showed nodular high signals on DWI, and the internal signals were relatively uniform. Thirty-one nodular goiters (Fig. 4) and nine Hashimoto’s thyroiditis cases showed slightly higher nodular signal intensity, as did 11 thyroid adenomas.

In 137 nodules, 77 were malignant (73 consistent with pathological findings) and 60 were benign (47 consistent with pathological findings). The sensitivity, specificity, and accuracy of MRI differential diagnosis were 84.9, 92.2, and 87.6% respectively.

The TI-RADS classification method was used in the ultrasound diagnosis report (grade 1–3a was benign and grade 3b–5 was malignant). Among 137 nodules, 88 were malignant (78 consistent with pathological findings) and 49 were benign (41 consistent with pathological findings). The sensitivity, specificity, and accuracy of ultrasound diagnosis were 90.1, 80.4, and 86.9% respectively. Finally, the qualitative diagnostic results of MRI and ultrasound were compared by chi-square test. The utility of these modalities for diagnosing thyroid nodules is compared in detail in Table 2.

**Table 2:** Comparison of sensitivity and specificity of MR and ultrasound in differential diagnosis of benign and malignant thyroid nodules (%)

Inspection mode	Sensitivity	Specificity	Accuracy
MRI (ADC)	84.9%(73/86)	92.2% (47/51)	87.6%
Ultrasound	90.1% (18/86)	80.4% (41/51)	86.9%
χ^2^	0.44	0.82	31.2
P	>0.05	>0.05	<0.05

## Discussion

In this study, 15 cases of malignant nodules were associated with Hashimoto’s thyroiditis and 2 cases with nodular goiter. Noda et al. [5] reported a statistical difference between benign and malignant thyroid nodules on T2SIR (*P* < 0.001), and the authors suggested that dense fibrous tissue in papillary carcinomas led to a decrease in T2SIR. In this study, we found no significant difference between T1 and T2SIR (*P* > 0.05), and the SIRs of benign and malignant nodules were smaller than those reported previously, which may be related to the type of machine or the type of samples. At present, there are few studies on the SIR, so it is premature to use T2SIR as a stable index for thyroid nodule identification.

Nodules were characterized by differential lobes or irregular margins, microcalcification, or mixed calcification based on the TI-RADS grading method [6]. Cheng et al. [7] reported that the blood flow characteristics of nodules could be used as an important reference index for differentiating benign and malignant nodules. However, there were some overlaps between benign and malignant nodules on sonograms, and previous studies suggested [5, 8] that the TI-RADS classification is still controversial. Malignant nodules have blurred boundaries, solid hypoechoic interiors, gravel-like calcification, and mixed internal blood flow, while lymphoma nodules have abundant internal blood flow signals. Ota et al. [9] reported that an enhanced posterior echo of nodules is an important differentiating sign of lymphoma compared to other malignant thyroid tumors; benign nodules have clear boundaries, low-signal and isoechoic interiors, and may be accompanied by coarse calcium. Most of the lesions had complete capsules, with rare blood flow signals in the nodules. Circular blood flow signals could be seen around the nodules, while blood flow was abundant in Hashimoto’s thyroiditis nodules. In this study, the sensitivity of ultrasound diagnosis of nodules was high (90.1%), but the specificity was not (80.4%). Compared with MRI, ultrasound is more sensitive for detecting micro-lesions and identifying micro-calcification. Almost all calcifications were detected. However, due to inherent limitations of ultrasound technology, the exploration of deep cervical tissue and the evaluation of peripheral lymph node metastasis are inferior to MRI.

The use of DWI has been increasingly studied for the differential diagnosis of benign and malignant thyroid nodules. The ADC value is related to the free movement of water molecules but is also affected by microcirculation perfusion. Wang et al. [10] concluded that the ADC value is an independent measurement factor in the differential diagnosis of benign and malignant thyroid nodules in multi-parameter DWI studies. We found that the ADC values of benign thyroid nodules were significantly higher than those of malignant thyroid nodules, which was consistent with previous studies [3, 10, 11]. Wu et al. [11] reported that DWI can provide ample information about tissue microstructure and physiological processes. The existence of organelles and biomacromolecules in tissues limits the free movement of extracellular water molecules. Conversely, Schueller-Weidekamm et al. [12] suggested that ADC values in malignant nodules were higher than those in benign nodules. The authors of that study proposed that increased follicular production in malignant nodules increases ADC values. In this study, the ADC value of malignant nodules (0.89 ± 0.21 × 10^− 3^ mm^2^/s) was lower than that of benign nodules (1.36 ± 0.17 × 10^− 3^ mm^2^/s). The main reason was that the number and density of malignant nodules were more than that of benign nodules, which resulted in decreased intercellular space. At the same time, the malignant nodules were mainly papillary carcinomas, which have increased mitosis and a higher nuclear-cytoplasmic ratio, corresponding to limited intracellular water molecule activity. Fibrosis also hampers the diffusion of water molecules to varying degrees, which is consistent with Cheng et al. [7]. Liu et al. [13] . suggested that irregular nodule margins are a strong predictor of malignant tendency. Malignant nodules on DWI usually have high signal intensity. Quantitative ADC values obtained from post-processing can objectively and qualitatively evaluate nodules. Using 1.12 × 10^− 3^ mm^2^/s as the threshold, we found higher specificity of DWI diagnosis (92.2%) and lower sensitivity (84.9%). Compared with ultrasound, the detection rate of lymph node metastasis was higher. According to Abdel Razek et al. [14], DWI combined with ADC maps can help identify metastatic non-necrotic lymph nodes and provide microscopic information about lymph node status. Recently, Wang et al. [15] concluded that a high b value (2000 s/mm^2^) has high accuracy for the differential diagnosis of benign and malignant thyroid micronodules.

Our results should be interpreted in the context of several limitations. Firstly, it was a retrospective study with relatively small number of patients, the preponderance of malignant nodules in the sample selection will inevitably affect some quantitative parameters of DWI. Secondly, the malignant group only included papillary, follicular, and lymphoma subtypes. Thirdly, we adopted b values of 0 and 1000 s/mm^2^ for DWI. A b values of 800 s/ mm^2^ was more commonly used in previous thyroid studies. Finally, ultrasound examination results were based on the opinion of one diagnostic physician, which is subjective. Therefore, we will continue to expand the sample sizes and types, perform comparative analysis with ultrasound, and conduct an in-depth comparative study of differences between groups.

## Conclusions

The threshold of the ADC value for differentiating benign and malignant thyroid nodules was 1.12 × 10^− 3^ mm^2^/s. There was no statistical difference in sensitivity or specificity between ultrasound and DWI. Ultrasound is superior to DWI for detecting calcification, while MRI is more useful for diagnosing lymph node metastasis. Both modalities play important roles in thyroid nodule diagnosis.

## Data Availability

The dataset supporting the conclusions of this article is available upon request to the corresponding author.

## Abbreviations

ADC: Apparent diffusion coefficient
AUC: Area under the ROC curve
DWI: Diffusion-weighted imaging
FOV: Field of view
MRI: Magnetic resonance imaging
ROC: Receiver operating characteristic
ROI: Region of interest
SIR: Signal intensity ratio
TE: Echo time
TI-RADS: Thyroid imaging reporting and date system
TR: Repetition time
TSE: turbo spin echo
